# Bovine Colostrum Whey Protein Hydrolysate Inhibits Cell DNA Damage and LDL Oxidation In Vitro

**DOI:** 10.3390/molecules22030456

**Published:** 2017-03-13

**Authors:** Shu-Hua Chiang, Shiu-Yu Wang, Chi-Yue Chang, Chih-Wei Chen

**Affiliations:** 1 Department of Health and Creative Vegetarian Science, FoGuang University, No. 160, Linwei Rd., Jiaosi, Yilan County 26247, Taiwan; shchiang@mail.fgu.edu.tw; 2 Department of Biological Science and Technology, I-Shou University, Kaohsiung 84001, Taiwan; ivy2533@yahoo.com.tw; 3 Department of Health Food, Chung Chou University of Science and Technology, Changhua 51591, Taiwan; charles0201@dragon.ccut.edu.tw

**Keywords:** bovine colostrum, whey protein hydrolysate, DNA oxidative damage, low-density lipoprotein (LDL), LDL oxidation

## Abstract

Whey protein isolated from bovine colostrums collected on the second day postpartum was two-stage hydrolyzed by alcalase and flavourzyme. The whey hydrolysates were finally fractionated by ultrafiltration (UF) with a 10 kDa molecular weight (MW) cutoff membrane and subsequently used to evaluate the effect of whey protein hydrolysis on inhibition of DNA oxidative damage and low-density lipoprotein (LDL) oxidation in vitro. Results showed that whey hydrolysis exhibited not only higher inhibitory activities of oxidative damage of deoxyribose but also an inhibitory effect on the breakdown of supercoiled DNA into open circular DNA and linear DNA. The quantities of 8-hydroxy-2′-deoxyguanosine (8-OH-2′-dG) formed with the addition of whey hydrolysate protein, the hydrolysate fraction of MW >10 kDa, and the hydrolysate fraction of MW <10 kDa were 0.25, 0.06, and 0.09 μg/mL, respectively. The lag time of conjugated diene formation of the control sample, which was only combined with cupric ions and LDL, was 90 min. The samples added with the hydrolysate fractions exhibited higher inhibitory activity on LDL oxidation. The whey hydrolysate fractions extended the lag time of conjugated diene formation to 270 min. The lag time of the whey hydrolysate fractions was 3 times that of the control.

## 1. Introduction

Bovine colostrum is the first milk produced postpartum and is typically defined as the first six postpartum milkings collected during the period of transition from colostrums to milk [1,2]. Several researchers have compared the composition of colostrums with those of mature milk and concluded that colostrums have higher levels of protein, lower levels of fat, a lactose solution rich in immunoglobulins, and other important immune elements and mediators [3,4,5]. Bovine colostrums are rich in cytokines, including interferon-γ (IFN-γ), interleukin (IL) and tumor necrosis factor-α (TNF-α), which are produced in the mammary gland and secreted after parturition [6]. Colostrums play a key role in the transmission of necessary nutrients, growth factors, and immunological components from mother to neonate. A broad range of animal and plant protein hydrolysates have been applied in the cosmetics and healthcare fields.

Whey proteins are globular proteins that are soluble over a broad pH range, and belong to byproducts of casein. The major constituents of bovine colostrum whey protein are α-lactalbumin (15%–20%) and β-lactoglobulin (55%–65%). The minor constituents include immunoglobulins (9%), bovine serum albumin (5.5%), lactoferrin, phospholipoproteins, bioactive factors, and enzymes [7]. Several functional and biological activities of bovine colostrum whey protein and whey protein hydrolysates have been reported, such as U937 cell growth inhibition and immunomodulatory activities [1], antioxidation activities [8], inhibition of angiotensin I-converting enzyme and downregulation of fatty acid synthesis in liver [7], and inhibition of DNA oxidative damage and LDL oxidation [2]. Protein hydrolysate is a mixture of proteoses, peptones, peptides, and free amino acids. It is possible, depending on enzyme specificity and the degree of hydrolysis (DH) achieved, to generate hydrolysate products exhibiting enhanced or reduced functionality.

DNA, as the repository of genetic information in living cells, is remarkably susceptible to damage induced by exogenous and/or endogenous factors [9]. In recent years, the damage degree of DNA induced by reactive oxygen species (ROS)—such as hydroxyl radicals (·OH), hydrogen peroxide (H_2_O_2_), and superoxide (O_2_^−^)—or the capacity of antioxidants for scavenging ROS have been widely investigated in the food technology and human health fields [9,10]. Several research papers indicate that oxidized low-density lipoprotein (LDL) within the arterial wall promotes the development of atherosclerosis [11]. Protection against LDL oxidation is an effective strategy to prevent atherosclerosis [11,12], and growing evidence from epidemiologic studies has shown that dietary antioxidants contribute to the prevention of coronary heart disease [11,13]. Many antioxidants have an effect on the inhibition of lipid oxidation. However, they fail to protect matrices such as DNAs, carbohydrates, and proteins from oxidative damage. For example, while butylated hydroxyanisole can effectively retard peroxidation of lipid, it is expected to result in pre-carcinoma of stomach in rats by an excessive intake, which is attributed to oxidative damage of DNA [14]. Therefore, efforts in searching for a bioavailable antioxidant to terminate chain reactions of radicals and to inhibit the oxidation of DNA in the human body have become critical.

The use of food protein hydrolysates is widely accepted in the field of cosmetics and healthcare products. Bovine colostrum protein hydrolysate products can be useful to enhance human health. However, to the best of our knowledge, bovine colostrum whey protein hydrolysates have not been the subject of study for inhibiting cell DNA damage and LDL oxidation. In this study, whey protein isolated from bovine colostrums collected on the second day postpartum was two-stage hydrolyzed by alcalase and flavourzyme to investigate the antioxidation of biomolecules, defense against DNA oxidative damage induced by hydrogen peroxide, and protection of oxidative modification of LDL. Furthermore, the possible relationship between cell DNA damage and LDL oxidation inhibitory activities and the hydrolysate fractions obtained by ultrafiltration with the molecular weight cutoff membranes was also evaluated.

## 2. Results

### 2.1. Degree of Hydrolysis of Whey Protein by Two-Stage Hydrolysis

**Figure 1:**
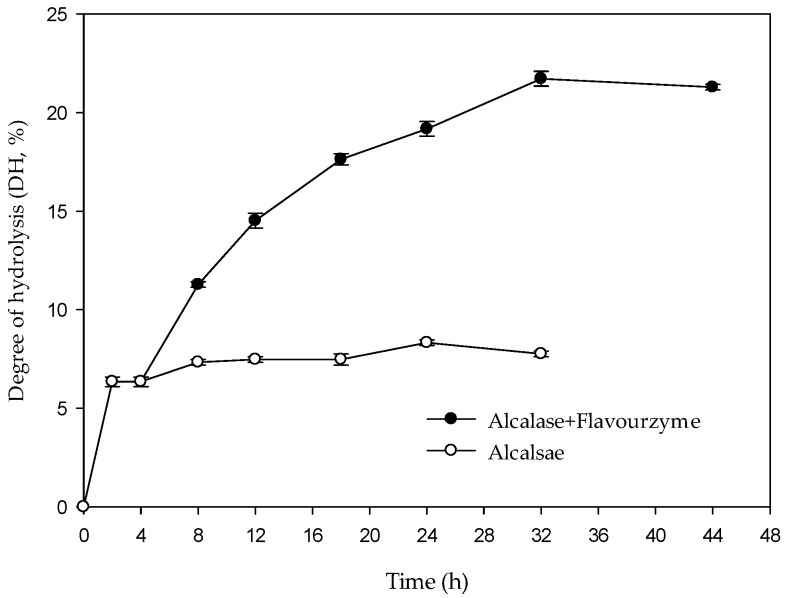
The degree of hydrolysis (DH) of whey protein hydrolysates during two-stage hydrolysis using alcalase and flavourzyme.

The two-stage hydrolysis of whey protein by alcalase and flavourzyme were monitored for up to 40 h. The hydrolysis curves are shown in Figure 1. The highest DH value was 8.32% in the first stage using alcalase hydrolysis (hydrolysis time 4 h; E/S = 2.0%). In the second stage, flavourzyme was added (hydrolysis time 40 h; E/S = 1.5%) and the highest DH value was 21.71%. From Figure 1, it is evident that DH increases with increasing enzyme concentration. The result is in agreement with the findings of Rebeca et al. (1991) [15], who reported that, for two-stage hydrolysis of fish protein, the DH increases with increasing enzyme concentration, and that DH is positively related to hydrolysis time.

### 2.2. Effect of Whey Protein Hydrolysate (WPH) and WPH Fractions on the Fenton Reaction-Induced Oxidative Damage of Deoxyribose

**Figure 2:**
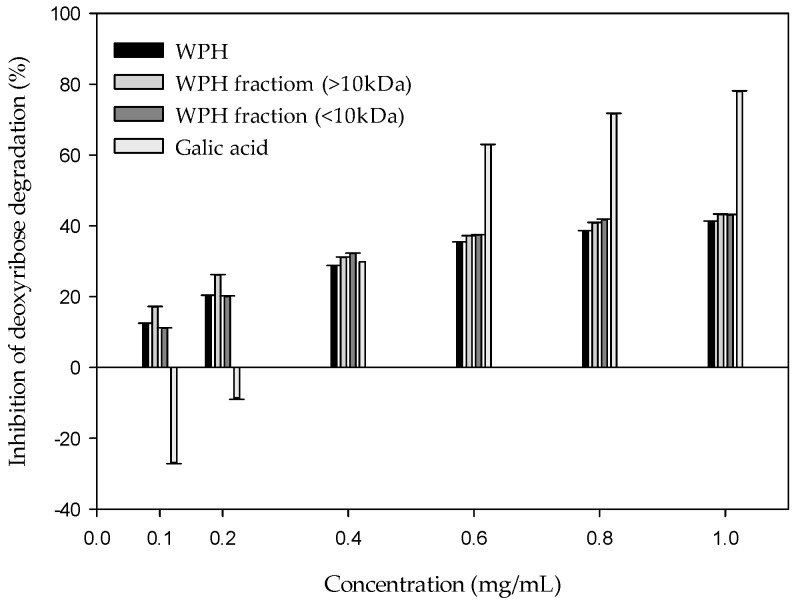
Effect of whey protein hydrolysate (WPH) and WPH fractions on the Fe^2+^–EDTA/H_2_O_2_/ascorbic acid (Asc)-induced oxidative damage of deoxyribose.

The result indicates that the WPH and WPH fractions have a significant inhibition against the oxidation damage of deoxyribose (Figure 2). Meanwhile, the inhibition rate goes up with the increasing sample concentration. When the concentration reaches 0.4 mg/mL, the inhibition rate of three samples become stable. The inhibition rates of WPH, WPH fraction (>10 kDa), and WPH fraction (<10 kDa) were 41.27%, 43.17%, and 43.09% at 1 mg/mL, respectively. In contrast, gallic acid shows oxidation promotion when the concentration is below 0.2 mg/mL. It is reported that gallic acid can clean O_2_^−^ and HOCl, and inhibits the peroxidation of liposome [16]. However, it promotes oxidation in the oxidation damage system of deoxyribose, induced by Fenton reaction. The concentration accords with the low level adopted in this experiment. The oxidative stability improves with increasing concentration. When the concentration is 1.0 mg/mL, the inhibition rate has reached 77.9%. This accords with Hsieh and Yen (2000) [17], which is 79% at 1.14 mg/mL. Chen et al. (2016) [2] reported the concentration as 1.0 mg/mL, with inhibition rates of whey, casein, and skimmed milk of 65.6%, 38.3%, and 66.9%, respectively. The result indicates that WPH and WPH fractions do not promote any oxidation. In contrast, they are good ·OH cleaners. WPH and WPH fractions demonstrate very good inhibition effects.

### 2.3. Effect of WPH and WPH Fractions on Single-Strand DNA Cleavage Induced by Fenton Reaction

**Figure 3:**
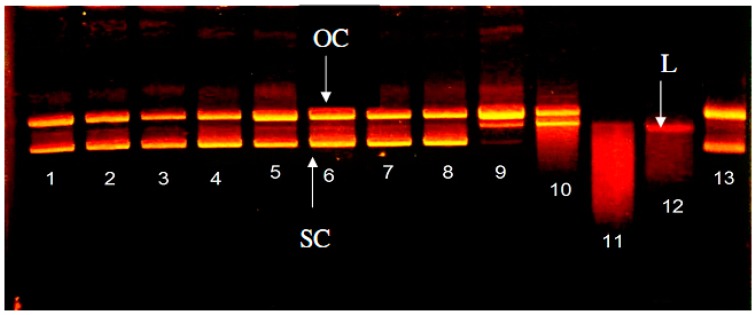
Effect of WPH and WPH fractions on single-strand DNA cleavage induced by Fe^2+^ and Fenton reaction. Phage ΨX174 supercoiled DNA (0.3 μg) was incubated with Fe^3+^ (lane 13); lanes 1–3: 0.1, 1.0, 10 mg/mL WPH; lanes 4–6: 0.1, 1.0, 10 mg/mL WPH fraction (>10 kDa); lanes 7–9: 0.1, 1.0, 10 mg/mL WPH fraction (<10 kDa); lanes 10–12: 0.1, 1.0, 10 mg/mL ascorbic acid.

Figure 3 shows that in the control group (lane 13), with only ferrous ions added, both supercoiled and open circular DNA can be found. There are free radicals generated in the Fenton reaction from supercoiled DNA. Some of the supercoiled DNA is cut into open circular DNA. This is similar to the results of Kobayashi et al. (1990) [18]. The concentration of 0.1 mg/mL (lanes 1, 4, 7) for the WPH and WPH fractions has no significant impact on the damage by ·OH. When the concentration is 1 mg/mL (lanes 2, 5, 8), the oxidation promotion can be observed in all solutions, and when the concentration is 10 mg/mL (lanes 3, 6, 9), the oxidation promotion disappears. The results showed that, at a low concentration, the WPH and WPH fractions have a limited ferrous ion chelating ability and cannot finish the ferrous ion chelating effectively. The reduction of oxidation damage is because of the chelating capacity, which increases when the concentration increases. For the ascorbic acid (Asc) (lanes 10–12), oxidation promotion can be observed at all concentrations, especially when the concentration is 1 mg/mL (lane 11). Worse DNA oxidation damage can be observed. As a result, no linear DNA can be seen and there are some DNA segments left.

### 2.4. Effect of WPH and WPH Fractions on the Oxidation of 2′-Deoxyguanosine (2′-dG) to 8-OH-2′-dG Induced by Fenton Reaction

In Table 1, it is shown that a significant difference can be found under different concentrations of WHP and WHP fractions. Compared to ascorbic acid, these solutions do not promote the generation of 8-OH-2′-dG under any circumstances. The output of WPH is a bit higher than the WHP fraction (>10 kDa) and WHP fraction (<10 kDa). A possible reason for this is that the WPH protein does not have a strong reducing capacity. Free radicals will not oxidize the 2′-dG continuously.

### 2.5. Effect of WPH and WPH Fractions on Bleomycin-Dependent DNA Damage

**Figure 4:**
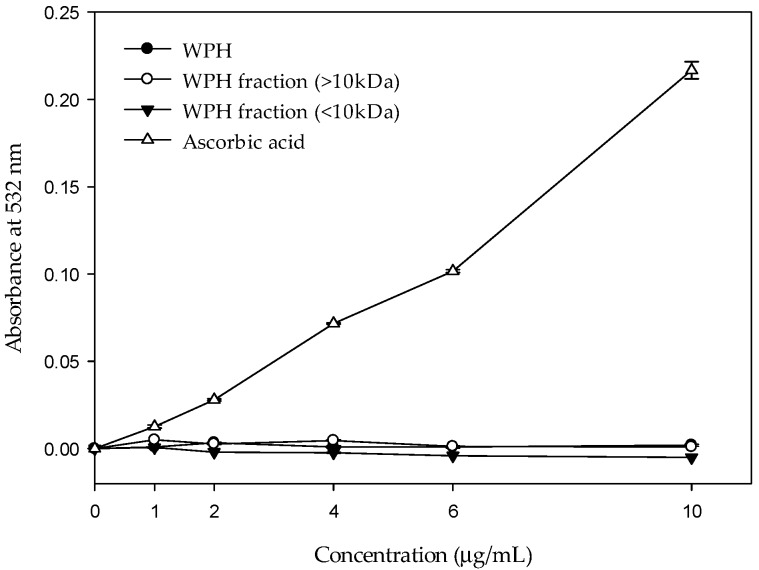
Effect of WPH and WPH fractions on DNA damage induced by bleomycin–Fe^3+^.

Figure 4 shows that under 10 μg/mL, the ascorbic acid promotes the oxidation damage of DNA by bleomycin–Fe^3+^ significantly. A major reason for this is that the ascorbic acid reduces the Fe^3+^ into Fe^2+^. The Fe^2+^ reacts with H_2_O_2_ and generates ·OH. Free radicals result in DNA damage [19]. The absorbance of WPH and WPH fractions does not increase with concentration in this system. A possible reason for this is that the WPH and WPH fractions are capable of cleaning ·OH and Fe^2+^ chelating. Meanwhile, none of them have the capacity to reduce the Fe^3+^ and protect the DNA from oxidation damage by free radicals.

### 2.6. The Protective Effects and Inhibition of Oxidative Damages of Biomolecules by WPH and WPH Fractions

Table 2 shows that the addition of WPH or WPH fractions at the initial stage of the reaction significantly reduced the oxidative damage caused by the addition of ascorbic acid at the later stage. The protective effect was 78.85% by WHP fraction (>10 kDa). The inhibition rates of DNA oxidation damage, induced by bleomycin–Fe^3+^/Asc, are 6.01%, 8.20%, and 6.01%, respectively, by treatment with WPH, WHP fraction (>10 kDa), and WHP fraction (<10 kDa) (Table 2). The inhibition rates of 8-OH-2′-dG generation from 2′-dG, induced by Fe^2+^–EDTA/H_2_O_2_/Asc, are 28.68%, 30.46%, and 30.76%, respectively (Table 2).

### 2.7. Effects of WPH and WPH Fractions on the Formation of Thiobarbituric Acid Reactive Substances (TBARS) and Conjugated Dienes on LDL Oxidation Induced by Cu^2+^

**Figure 5:**
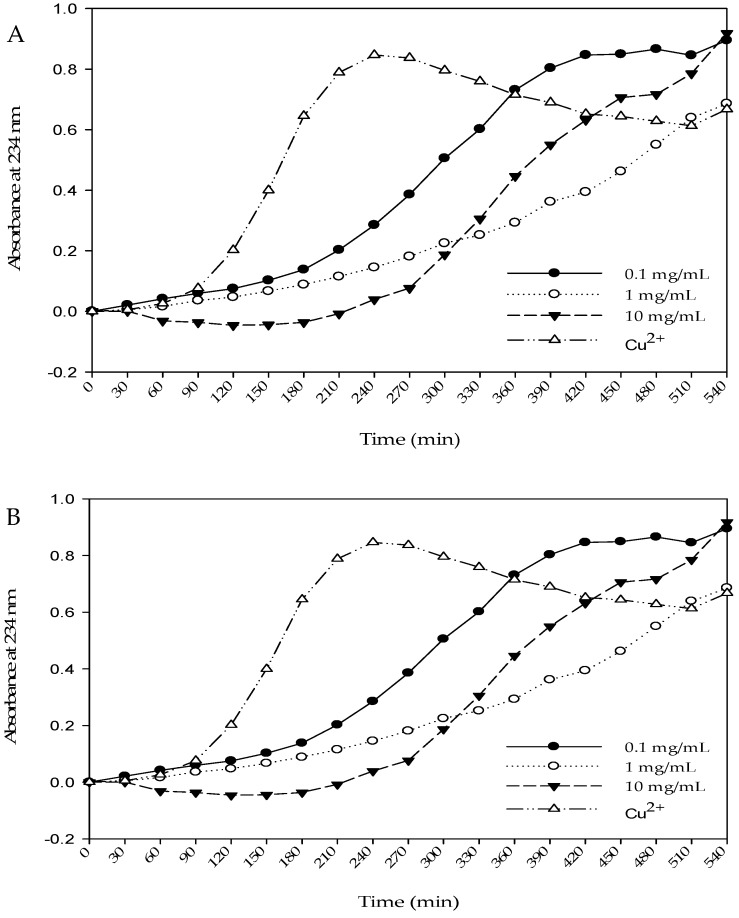

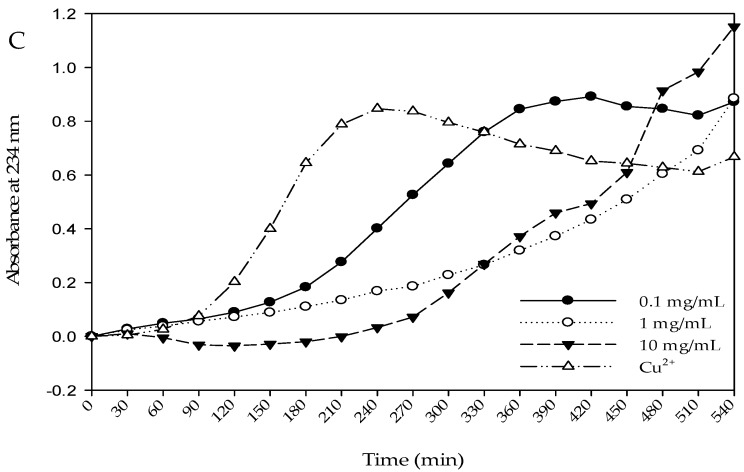
Effects of WPH and WPH fractions on Cu^2+^-mediated conjugated diene formation in low-density lipoprotein (LDL). LDL (100 μg protein/mL) was incubated with 10 μM CuSO_4_ at 37 °C in the absence or presence of WPH and WPH fractions. Conjugated diene was used to measure the absorbance at 234 nm every 30 min for 540 min and the results express relative absorbance at 234 nm. (**A**) WPH; (**B**) WPH fraction (>10 kDa); (**C**) WPH fraction (<10 kDa).

Table 3 shows the protection provided by WPH and WPH fractions against LDL oxidation damage. The results showed that when the concentration is 1 mg/mL, both WPH and WPH fraction (>10 kDa) have good oxidative stabilities. The TBARS outputs are 3.7 and 3.65 nM/mL, respectively, both significantly lower than the control group. Meanwhile, the TBARS output tends to increase with decreasing concentration. Table 3 also shows the effects of WPH and WPH fractions on the formation of conjugated diene from LDL oxidation induced by Cu^2+^. Only LDL and Cu^2+^ are added into the control group, whose lag time is 90 min. When the concentration is 0.1 mg/mL, the lag time for all groups is quite close to that of the control group. When the concentration is 1.0 mg/mL, the lag time for WPH and WPH fractions extends to 180 min (Figure 5). No significant extension is found for the rest of the groups. When the concentration is 10 mg/mL, significant inhibition can be observed. The lag times for WPH, WPH fraction (>10 kDa), and WPH fraction (<10 kDa) were 240, 270, and 270 min, respectively. They are 2.66, 3.0, and 3.0 times that of the control group.

## 3. Discussion

The first study tries to determine whether the WPH and WPH fractions can be used to limit the oxidation damage of deoxyribose induced by the Fe^3+^–EDTA/H_2_O_2_/Asc system. Figure 2 demonstrates the inhibition against the Fenton reaction-induced oxidation damage of deoxyribose by WPH and WPH fractions. Since the deoxyribose will break into malondialdehyde (MDA) by the attack of hydroxyl free radicals, the formation of one of the products, malondialdehyde, forms the basis of the deoxyribose assay. Damage to the sugar parts of the DNA structure can lead to breaks in the strands of DNA, because these constitute the phosphate–deoxyribose backbone [20]. The result indicates that the WPH and WPH fractions do not promote any oxidation. On the contrary, they are a good ·OH cleaner. WPH and WPH fractions demonstrate very good inhibition effects. This also proves that none of them have obvious reducing power [8]. Kim et al. (2013) [7] reported that whey protein possesses antioxidant activity, which has been recognized as the factor responsible for chelating of transition metals by serum albumin and lactoferrin, an iron-binding glycoprotein, as well as for the free radical-scavenging activity shown by amino acids such as Try and Cys. Bayram et al. (2008) [21] reported that the WPH contains immunoglobulins (Ig), bovine serumal bumin (BSA), β-lactoglobulin (β-Lg), α-lactalbumin (α-La), lactoferrin, and peptides. The WHP fractions are richer in free amino acids (e.g., Met, Cys, and His) and small peptides. It may be the reason that WPH fractions provide strong reducing activity to scavenging free radicals—more so than WPH. Peptides from protein hydrolysates are reported to act as antioxidants through mechanisms of inactivation of reactive oxygen species (ROS), free radical-scavenging, inhibition of lipid peroxidation, chelation of metal ions, or a combination of these mechanisms. The major mode of action derives from the inherent amino acid composition and sequence of a peptide. Brandelli et al. (2015) [22] reported that whey protein hydrolyzed by different proteases—namely trypsin, pepsin, alcalase, promatex, flavourzyme, or protease N. The hydrolysate generated by Alcalase 2.4 L showed the highest antioxidant activities, and seven different peptides showing strong antioxidant activities were isolated. The antioxidant peptide WYSL displayed the highest 2,2-diphenyl-1-picrylhydrazyl (DPPH) radical-scavenging activity and superoxide radical-scavenging activity.In a study on the extractions of *Eucommia* bark, *Eucommia* bark leaf and fried and fresh *Eucommia* barks had inhibition effects of 85%, 68%, and 49%, respectively. That accords with our result. Furthermore, the inhibition rate tends to increase with the concentration increase [17]. Although the reducing power plays an important role in oxidative stability, it is even more important in oxidation promotion, especially when there is a transition metal. The reducibility may promote the oxidation damage of the biological molecules [20]. Strong reducing power can reduce Fe^3+^ into Fe^2+^ and increase the formation of ·OH. That is why gallic acid fails to keep Fe^2+^ away from the biological molecules, together with its poor Fe^2+^-chelating ability. As a result, Fe^2+^ cannot be controlled, and there are site-specific oxidation damages on biological molecules [14].

ΨX174 RF I is a supercoiled double-stranded DNA. If a single strand of ΨX174 RF I DNA is broken, it switches from type replicative form (RF) I into type RF II, which is not supercoiled. The supercoiled architecture is then turned into open circular. The supercoiled DNA has a good electrophoretic fluidity in colloid. The double-strand structure may break into open circular DNA, which will lower the electrophoretic fluidity. Linear DNA has an electrophoretic fluidity between supercoiled and open circular DNA [23]. Figure 3 shows the ascorbic acid (lanes 10–12) oxidation promotion can be observed in all concentrations, especially when the concentration is 1 mg/mL (lane 11). Worse DNA oxidation damage can be observed. As a result, no linear DNA can be seen and there are some DNA segments left. The study pointed out that with 5 mM (0.88 mg/mL) ascorbic acid, the DNA can be cut into drags. That accords with our results [24]. Although the ascorbic acid and polyphenol compounds can be used to control the overoxidation of the lipids, it is found that these substances are oxidation-promotional. For example, the ascorbic acid can prevent the cells from oxidation damages, but if Fe^3+^ or Cu^2+^ are present, generation of reactive oxygen species (ROS) will be promoted [25]. They also promote the generation of 8-OH-2′-dG in mouse cystoblasts [26].

8-OH-2′-dG turns out to be the most significant biological index of DNA oxidation damage in both internal and external experiments [27,28]. At the same time, it has the potential to cause mutagenicity [29]. Therefore, measuring the content of 8-OH-2′-dG can be used to evaluate the oxidation pressure in the body [30]. According to the results (Table 1), massive generation of 8-OH-2′-dG is impossible.

Yen et al. (1997) [31] pointed out that adding ascorbic acid into the system at a similar concentration can result in the transformation of 2′-dG into 8-OH-2′-dG. That accords with the results of this experiment. In a study on bovine colostrums protein, its 8-OH-2′-dG output was higher than that of WPH and WPH fractions [2].

Bleomycin is a member of the glycopeptide family. It is a kind of antibiotic containing several rare amino acid and carbohydrate molecules. The type differs based on the terminal amino acid. Bleomycin is a mixture [32] of bleomycin adopted in a clinic and includes 55%–70% A2, 25%–32% B2, A20 (<7%), and 1% B4. Its cell base looks radial because of toxic effects. When there is Fe^2+^ and oxygen, the DNA will be destroyed and the thymine will become dissociative, or the DNA structure will be broken [33].

Figure 4 shows that the absorbance of WPH and WPH fractions does not increase with concentration. A possible reason for this is that the WPH and WPH fractions are capable of cleaning ·OH and chelating Fe^2+^.

Table 2 shows the protective effect of WPH and WPH fractions on 2′-dG. The results showed that the addition of WPH or WPH fractions at the initial stage of the reaction significantly reduced the oxidative damage caused by the addition of ascorbic acid at the later stage. The protective effect was 78.85% by WHP fraction (>10 kDa) and the protective effect of the samples were in an order of the WHP fraction (>10 kDa) > WHP fraction (<10 kDa) > WPH (Table 2). From the results of 8-OH-2′-dG generation and DNA oxidation damage induced by the bleomycin–Fe^3+^ system, the ascorbic acid may result in significant oxidation damage with the existence of Fe^3+^. In this experiment, we added the ascorbic acid into the reaction system first and waited for the oxidation damage. After that, WPH and WPH fractions were added to it. Then, comparison of the inhibition of the ascorbic acid was made and inhibition rates were calculated. For the impacts of WPH and WPH fractions on DNA oxidation damage induced by bleomycin–Fe^3+^/Asc, the inhibition rates are 6.01%, 8.20%, and 6.01%, respectively (Table 2). With the exception of WPH and WPH fractions, significant inhibitions were demonstrated. For the impacts of WPH and WPH fractions on 8-OH-2′-dG generation from 2′-dG induced by Fe^2+^–EDTA/H_2_O_2_/Asc, the inhibition rates are 28.68%, 30.46%, and 30.76%, respectively (Table 2). WPH fractions are better than WPH in 8-OH-2′-dG inhibition. Chen et al. (2016) [2] reported that when testing the effect of whey, casein, and skimmed milk on 8-OH-2′-dG generation from 2′-dG, induced by Fe^2+^–EDTA/H_2_O_2_/Asc, they found inhibition rates of 20.01%, 18.18%, and 11.07%, respectively. Chiang and Chang (2005) [8] reported that lactoferrin has strong antioxidant activity such as reducing power, Fe^2+^ chelation, and free radical-cleaning ability; the content of lactoferrin was in order of whey > casein > skimmed milk. Based on the above results, we come to the conclusion that the inhibition against oxidation damage on biological molecules is likely to be: whey > casein > skimmed milk. High Fe^2+^ chelating activity and free radical cleaning are reasons for the high inhibition by whey protein [8].

Table 3 shows the effects of WPH and WPH fractions on the formation of malondialdehyde (MDA) from LDL oxidation induced by Cu^2+^. MDA is one of several low-molecular-weight end products formed via the decomposition of certain primary and secondary lipid peroxidation products. At low pH and elevated temperature, MDA readily participates in a nucleophilic addition reaction with 2-thiobarbituric acid (TBA), generating a red, fluorescent 1:2 MDA:TBA adduct. The thiobarbituric acid reactive substances (TBARS) assay measures substances produced by oxidation that were able to react with thiobarbituric acid. MDA is one of these substances from the TBARS. Decreased production of TBARS indicates that the samples can effectively inhibit LDL oxidation.

The results are shown in Table 3. The quantity of TBARS was 5.13 nM/mL in control groups. The quantity of TBARS of WPH and WPH fractions groups were lower than control group and the inhibition effect of TBARS formed with the WHP fraction (>10 kDa) was better than the other two. On the contrary, the TBARS output tends to increase with increasing concentration by addition WHP fraction (<10 kDa). We speculate that the cause of this is that the major constituents of the WHP fraction (<10 kDa) were peptides and free amino acids. Free radicals attack amino acids and form new free radicals (carbon-centered alkyl radicals, R·); then, the amino acid radicals and oxygen molecules react to form peroxide radicals of amino acids (ROO·), resulting in more lipid oxidation and TBARS formation. Therefore, the higher concentration of WHP fraction (<10 kDa) has more free amino acids, leading to more lipid oxidation. Zhang and Omaye (2001) [34] reported that β-carotene increased with increasing of TBARS concentration, which was mainly due to the formation of β-carotene–OO· at high concentration. This is related to the WHP fraction (<10 kDa) lipid oxidative damage. Table 3 also shows the effects of WPH and WPH fractions on the formation of conjugated diene from LDL oxidation induced by Cu^2+^. If the sample has the inhibition effect of LDL oxidation, the lag phase time will increase. Table 3 shows that the formation of conjugated dienes could be inhibited by treatment with WPH and WPH fractions. The WPH fractions extended the lag time of conjugated diene formation to 270 min.

## 4. Materials and Methods

### 4.1. Materials

The bovine colostrums used in this study were collected from a cow on its second postpartum day at the Chu-En Ranch, Hsiushui, Changhua, Taiwan.

### 4.2. Preparation of Whey Proteins

The colostrums were collected at approximately 8 a.m. and promptly centrifuged at 10,000× *g* for 30 min at 4 °C to remove fat. Subsequently, the colostrums were adjusted to pH 4.6 using 1.0 N HCl and kept in a water bath at 30 °C for 30 min to complete the precipitation of caseins. The supernatant thus obtained was adjusted to pH 7.0, using 1.0 N NaOH, and centrifuged again at 10,000× *g* for 30 min at 4 °C. The final supernatant after the two centrifugations was treated as a whey protein. The skimmed milk, caseins, and whey proteins were freeze-dried and stored at −20 °C until used.

### 4.3. Enzymatic Hydrolysis

Two gram of whey protein (WP) sample was dissolved in 100 mL of distilled water and stirred with a stirrer for 10 min at room temperature. The reaction pH levels were adjusted to pH 8.5 for alcalase hydrolysis (E/S = 2.0%; 50 °C) and pH 7.5 for flavourzyme hydrolysis (E/S = 1.5%; 40 °C) with 1 N NaOH or HCl. In the two-stage hydrolysis process, alcalase (E/S = 2.0%) was used in the first stage and the hydrolysis time was 4 h. Flavourzyme (E/S = 1.5%) was used in the second stage and the hydrolysis times were 4, 8, 12, 20, 28, and 40 h. Two-stage hydrolysis was stopped by water bath at 90 °C for 15 min. DH of the whey protein hydrolysate (WPH) was calculated as (amino nitrogen/total nitrogen) × 100%, where the total nitrogen and amino nitrogen contents were determined by the semi-micro-Kjeldahl method and the formol titration method AOAC (2006) [35], respectively.

### 4.4. Ultrafiltration (UF)

The WPH was fractionated using a UF system with a molecular weight cutoff (MWCO) (10 kDa). The WPH fraction was divided into two parts designated as >10 and <10 kDa, which were lyophilized and stored at −20 °C until use.

### 4.5. Effect of WPH and WPH Fractions on Deoxyribose Damage (Fenton Reaction)

To test the ability of the WPH and WPH fractions to inhibit oxidative damage of deoxyribose, the Fenton reaction model system, which contained FeCl_3_–EDTA and H_2_O_2_, was used with the method of Smith et al. (1992) [19]. The reaction mixture (3.5 mL), which contained the sample (0.1–1.0 mg/mL), deoxyribose (3 mM), H_2_O_2_ (1 mM), potassium phosphate buffer (20 mM, pH 7.4), FeCl_3_ (50 μM), and EDTA (100 μM), were incubated at 37 °C for 1 h with the addition of ascorbic acid (100 μM). The extent of deoxyribose degradation was measured using the TBARS method [36]. One milliliter of 1% TBA and 1 mL of 2.8% trichloroacetic acid (TCA) were added to the mixture, which was then heated in a water bath at 100 °C for 20 min. The absorbance of the resulting solution was measured by spectrophotometer at 532 nm. The control sample did not have WPH or WPH fraction solutions.

### 4.6. Effect of WPH and WPH Fractions on DNA Damage (Fenton Reaction)

To study the effect of bovine colostrums protein on the Fenton-induced oxidative breakage of supercoiled DNA to open circular DNA, DNA electrophoresis was employed using the method of Kobayashi et al. (1990) [18] with modification. WPH and WPH fractions samples (0.1, 1, 10 mg/mL), 0.3 μL of DNA (1 μg/μL), 0.1 M sodium phosphate buffer (pH 7.4), and FeCl_2_ (50 μM) were mixed in a plastic tube to get a final volume of 10 μL. The mixture was incubated at 37 °C for 1 h. The reaction was stopped by the addition of 5 μL of 0.1 M EDTA containing 50% (*w*/*v*) sucrose and 0.1% bromophenol blue, and the solution was subjected to electrophoresis in 0.7% agarose gels with 40% mM Tris-HCl/5 mM sodium acetate/1 mM EDTA as the running buffer. The gel was stained with ethidium bromide (0.05 mg/L), viewed, and photographed on a transilluminator. A scanner (ARTEC, Taichung, Taiwan) scanned the photographic negatives in order to quantify the relative amount of DNA in each band, using the ID Image Analysis Software (version 2.02, Kodak Digital Science, Rochester, NY, USA).

### 4.7. Effect of WPH and WPH Fractions on Oxidation of 2′-Deoxyguanosine (Fenton Reaction)

The effects of WPH and WPH fractions on the oxidation of 2′-deoxyguanosine (2′-dG) to 8-hydroxy-2′-deoxyguanosine (8-OH-2′-dG) were assayed, using the method of Yen et al. (1997) [29] with modification. The reaction mixture (1.4 mL), containing WPH and WPH fractions samples (1, 2, 4, 6, 8, 10 mg/mL), 2′-dG (0.5 mM), and potassium phosphate buffer (20 mM, pH 7.4), was initiated using the Fenton reaction model system (H_2_O_2_ (50 mM), FeCl_3_ (1.3 mM), and EDTA (6.5 mM)) with the addition of ascorbic acid (15 mM). The entire mixture was incubated at 37 °C for 30 min, and incubation as terminated by placing the samples in an ice-bath, and then filtered through a 0.45 μm filter before use. The filtrate was analyzed by HPLC (Hitachi, Tokyo, Japan), using a LiChrosphere RP-18 column 150 mm × 4 mm, 5 μm) and UV detector (measured at 254 nm). The mobile phase contained 6.5% ethanol in 50 mM phosphate buffer, and the flow rate was 0.5 mL/min. 2′-dG and 8-OH-2′-dG were identified through comparison of their retention times with those of known standards, and the amount of 8-OH-2′-dG was determined based on the peak areas in the chromatograms.

### 4.8. Effect of WPH and WPH Fractions on Bleomycin-Dependent DNA Damage

The influence of WPH and WPH fractions on bleomycin-dependent DNA damage was determined according to the method of Aruoma et al. (1993) [16]. A solution (3.5 mL), which contained WPH and WPH fraction samples (1, 2, 4, 6, 10 μg/mL), calf thymus DNA (0.2 mg/mL), bleomycin (0.05 mg/mL), potassium phosphate buffer (20 mM, pH 7.4), FeCl_3_ (25 μM), and MgCl_2_ (5 mM), was incubated at 37 °C for 1 h with or without the addition of ascorbic acid (240 μM). A portion (0.1 mL) of EDTA (100 mM) was added to the mixture, which was then measured using the TBA method as described above for the assay of deoxyribose damage.

### 4.9. Inhibition of Oxidative Damage of Biomolecules by WPH and WPH Fractions

To assess the effect of WPH and WPH fractions on the inhibition of oxidative damage of biomolecules, two experiments were designed, one for the oxidation of 2′-dG induced by Fenton reaction and the other for the oxidation of DNA induced by bleomycin–Fe^3+^ (1.5 mM) and ascorbic acid (10 μg/mL), which were applied to the reaction solutions specified in Section 2.6 and Section 2.7 at the beginning of reaction, respectively, as the simulator of oxidative damage. Upon halfway through the reaction (10 mg/mL) time, bovine colostrum proteins (10 μg/mL) were added to retard the oxidation reaction, respectively. The resulting reaction solutions were then analyzed at the end of the reaction using the same procedures as described in Section 4.7 and Section 4.8.

### 4.10. LDL Preparation

Fasting plasma, for LDL isolation, was collected from normal human volunteers in tubes containing ethylenediaminetetraacetic acid (EDTA; 1 mg/mL). LDL (100 μg protein/mL) was isolated by sequential ultracentrifugation using a Hitachi ultracentrifuge (Himac CS 120GX, Hitachi) as described by Chen et al. (2016) [2] with a minor modification. LDL solution was flushed with N_2_ gas, stored at 4 °C, and used within 1 week after preparation. Protein was measured using a Bio-Red kit, with bovine serum albumin as a standard. For oxidation experiments, LDL was dialyzed three times against 1 L (1000-fold volume) of phosphate-buffered saline (PBS, containing 0.01 M phosphate-buffer and 0.15 M NaCl, pH 7.4) in the dark at 4 °C for 24 h.

### 4.11. LDL Oxidation

Dialyzed LDL (100 μg protein/mL) was diluted in 10 mM PBS and incubated at 37 °C in the presence or absence of 10 μM CuSO_4_. Oxidation was performed with or without the sample solution of WPH and WPH fractions. After incubation, lipid peroxidation of the LDL was measured as described below. Ascorbic acid oxidation was used for reference.

### 4.12. Thiobarbituric Acid Reactive Substances (TBARS)

TBARS were measured using the method described by Yagi (1989) [37]. LDL solution (0.10 mg protein/mL) was added with various concentrations of colostrums proteins specified as 0.001, 0.01, 0.1, and 1.0 mg/mL. After 1 h standing in room temperature, a mixture containing Cu^2+^ solution (10 mM) was added and the solution stood for another 24 h in a water bath at 37 °C for completion of reaction. At the end, 0.2 mL reaction solution was taken and combined with 0.2 mL TCA (20% *w*/*v*) and 0.2 mL TBA (0.67% *w*/*v* dissolved in 0.3% NaOH solution). After thorough mixing of the reaction solution, it was heated in a water bath at 90–95 °C for 45 min. A spectrofluorometer (Hitachi F-3010) was applied to determine Ex/Em: 532 nm/600 nm. Using 1,1,3,3-tetramethoxypropane as standard for the calibration curve, the content of TBARS could be calculated.

### 4.13. Conjugated Diene

Conjugated diene formation was measured by determining the absorbance increase at 232 nm of LDL solution (100 μg·protein/mL) in PBS incubated with CuSO_4_ (10 μM) in the absence or presence of various concentrations of WPH and WPH fractions (0.01, 0.1, and 1 mg/mL). The absorbance was measured every 30 min for 540 min using a Hitachi U-2000 spectrophotometer, and the results were expressed as relative absorbance at 234 nm. The duration of the lag phase was calculated by extrapolating from the propagation phase.

### 4.14. Statistics

Results in this study were analyzed by one-way ANOVA, correlation, and stepwise regression functions in SAS (SAS, 2001).

## 5. Conclusions

The results indicate that WPH and WPH fractions are not prooxidants. The decreasing sequence of inhibitive ability on oxidative damage of biomolecules is WHP fraction (>10 kDa) > WHP ≈ WHP fraction (<10 kDa). Recent reports on the clinical inspection of carcinoma, AIDS, and pneumonia have shown more and more specific evidence of the positive health effects from dietary intake of whey protein [38,39,40]. Although few researches have investigated the effect of whey protein on the body composition of humans, whey proteins have been demonstrably shown to promote glutathione content in various cells [41]. The reports regarding the treatment benefits of using bovine colostrum from antioxidant activity and composition were not substantial. Therefore, at present, results are based on the lactoferrin contents in bovine colostrums as an index of antioxidant activity: Chiang and Chang (2005) [8] reported that lactoferrin has strong antioxidant activity such as reducing power, Fe^2+^ chelation, and free radical-cleaning ability; the lactoferrin content was in the order whey > casein > skimmed milk. The lactoferrin contents of whey were significantly higher than casein and skimmed milk. In the present study, results showed that WPH and WPH fractions exhibited not only higher inhibitory activities of oxidative damage of deoxyribose, but also inhibitory effects on the breakdown of supercoiled DNA into open circular DNA and linear DNA. The quantities of 8-OH-2′-dG formed under WPH, WHP fraction (>10 kDa), and WHP fraction (<10 kDa) treatment were 0.25, 0.06, and 0.09 μg/mL, respectively. The quantity of malondialdehyde formed through LDL oxidation induced by cuprous ions was significantly decreased as WPH and WPH fractions were added, in which WHP fraction (>10 kDa) and WPH resulted in a more significant decrease than WHP fraction (<10 kDa). The formation of conjugated dienes could be inhibited by treatment with WPH and WPH fractions. WPH fractions exhibited the longest lag time of conjugated diene formation among the colostrum proteins. The lag time of the whey was 3 times that of the control. From these results, the WPH and WPH fractions have the potential to inhibit DNA oxidation damage and LDL oxidation. However, the mechanism and the functional composition, such as peptide sequence, remains unclear. Further purification of the bovine colostrum whey protein hydrolysate peptide sequence and investigations using clinical or animal models would be worthwhile.

## Figures and Tables

**Table 1:** Effect of WPH and WPH fractions on the oxidation of 2′-dG to 8-OH-2′-dG induced by Fenton reaction.

Addition to RM *	8-OH-2′-dG (μg/mL)		
	WPH	WPH Fraction (>10 kDa)	WPH Fraction (<10 kDa)
Blank (PBS)	0.47 ± 0.05 ^b,^**	0.47 ± 0.05 ^b,^**	0.47 ± 0.05 ^b,^**
15 mM ascorbic acid	10.24 ± 0.19 ^a,A^	10.24 ± 0.19 ^a,A^	10.24 ± 0.19 ^a,A^
1 mg/mL	0.25 ± 0.04 ^c,B^	0.06 ± 0.01 ^c,A^	0.09 ± 0.01 ^c,A^
2 mg/mL	0.12 ± 0.02 ^d,B^	0.04 ± 0.0 ^c,A^	0.06 ± 0.01 ^c,A^
4 mg/mL	0	0	0.03 ± 0.0 ^c^
6 mg/mL	0	0	0
8 mg/mL	0	0	0
10 mg/mL	0	0	0

* RM (reaction mixture) containing 0.5 mM 2′-dG, 1.3 mM FeCl_2_, 50 mM H_2_O_2_, 6.5 mM EDTA, 15 mM ascorbic acid, and 0.1 M phosphate buffer (pH 7.4) was shaken at 37 °C for 30 min. ** Values with different superscripts are significantly different (*p* < 0.05).

**Table 2:** Effect of WPH and WPH fractions on the DNA damage induced by bleomycin-Fe^3+^/Asc and oxidation of 2′-dG to 8-OH-2′-dG induced by Fe^2+^-EDTA/H_2_O_2_/Asc.

Addition to RM*	Protective Effect of 2′-dG		Bleomycin–Fe^3+^/Asc		Fe^2+^-EDTA/H_2_O_2_/Asc.	
	8-OH-2′-dG (μg/mL)	Inhibition (%)	Absorbance at 532 nm	Inhibition (%)	8-OH-2′-dG (μg/mL)	Inhibition (%)
Ascorbic acid	5.91 ± 0.28 ^a,^**		0.183 ± 0.01 ^a,^**		10.11 ± 0.51 ^a,^**	
WPH	1.77 ± 0.18 ^b^	70.05 ± 4.38 ^b^	0.172 ± 0.02 ^b^	6.01 ± 0.15 ^b^	7.21 ± 0.45 ^b^	28.68 ± 1.61 ^a^
WPH fraction (>10 kDa)	1.25 ± 0.19 ^c^	78.85 ± 4.09 ^a^	0.168 ± 0.0 ^b^	8.20 ± 0.62 ^a^	7.03 ± 0.45 ^b^	30.46 ± 1.90 ^a^
WPH fraction (<10 kDa)	1.81 ± 0.10 ^b^	69.37 ± 1.30 ^b^	0.172 ± 0.01 ^b^	6.01 ± 0.49 ^b^	7.00 ± 0.55 ^b^	30.76 ± 2.17 ^a^

*RM (reaction mixture) for DNA damage (containing 0.05 mg/mL bleomycin, 25 μM FeCl_2_, 5 mM MgCl_2_, 0.2 mg/mL calf thymus DNA, 30 mM phosphate buffer (pH 7.4), and 10 μg/mL ascorbic acid) was shaken at 37 °C for 30 min, then reacted with 10 mg/mL whey, casein, and skimmed milk for 30 min. ** RM (reaction mixture) for protective effect and 2′-dG to 8-OH-2′-dG (containing 0.5 mM 2′-dG, 1.3 mM FeCl_2_, 50 mM H_2_O_2_, 6.5 mM EDTA, 15 mM ascorbic acid, and 0.1 M phosphate buffer (pH 7.4) was shaken at 37 °C for 30 min.

**Table 3:** Effects of WPH and WPH fractions on the formation of thiobarbituric acid reactive substances (TBARS) and conjugated dienes on low-density lipoprotein (LDL) oxidation induced by Cu^2+^.

Concentration (mg/mL)	WPH	WPH Fraction (>10 kDa)	WPH Fraction (<10 kDa)	Concentration (mg/mL)	WPH	WPH Fraction (>10 kDa)	WPH Fraction (<10 kDa)
	TBARS (n mol/mL)				Lag Time * (min)		
Blank	5.13 ± 0.01 ^a,^**	5.13 ± 0.01 ^a,^**	5.13 ± 0.01 ^a,^**	Blank	90	90	90
0.001	4.98 ± 0.02 ^a^	4.65 ± 0.02 ^a,b^	4.10 ± 0.05 ^b^	0.1	180	180	180
0.01	4.82 ± 0.01 ^a^	4.22 ± 0.04 ^b^	4.22 ± 0.04 ^b^	1.0	210	210	210
0.1	4.19 ± 0.03 ^b^	3.89 ± 0.01 ^b,c^	4.85 ± 0.07 ^a^	10	240	270	270
1.0	3.70 ± 0.01 ^c^	3.65 ± 0.01 ^c^	4.84 ± 0.02 ^a^	-	-	-	-

* Conjugated diene formation was measured by determining the absorbance at 234 nm every 30 min for 540 min. ** Means with different letters within a row are significantly different (*p* < 0.05).
